# Reconstitution of Cytokinin Signaling in Rice Protoplasts

**DOI:** 10.3390/ijms22073647

**Published:** 2021-03-31

**Authors:** Eunji Ga, Jaeeun Song, Myung Ki Min, Jihee Ha, Sangkyu Park, Saet Buyl Lee, Jong-Yeol Lee, Beom-Gi Kim

**Affiliations:** Metabolic Engineering Division, National Institute of Agricultural Sciences, RDA, Jeonju 54874, Korea; vapofi@naver.com (E.G.); icanje@korea.kr (J.S.); mkmin66@korea.kr (M.K.M.); hjh10942@korea.kr (J.H.); psk2779@korea.kr (S.P.); buylee@korea.kr (S.B.L.); jy0820@korea.kr (J.-Y.L.)

**Keywords:** rice, protoplast, cytokinin, signaling, reconstitution

## Abstract

The major components of the cytokinin (CK) signaling pathway have been identified from the receptors to their downstream transcription factors. However, since signaling proteins are encoded by multigene families, characterizing and quantifying the contribution of each component or their combinations to the signaling cascade have been challenging. Here, we describe a transient gene expression system in rice (*Oryza sativa*) protoplasts suitable to reconstitute CK signaling branches using the CK reporter construct *TCSn:fLUC*, consisting of a synthetic CK-responsive promoter and the firefly luciferase gene, as a sensitive readout of signaling output. We used this system to systematically test the contributions of CK signaling components, either alone or in various combinations, with or without CK treatment. The type-B response regulators (RRs) OsRR16, OsRR17, OsRR18, and OsRR19 all activated *TCSn:fLUC* strongly, with OsRR18 and OsRR19 showing the strongest induction by CK. Cotransfecting the reporter with *OsHP01*, *OsHP02*, *OsHP05*, or *OsHK03* alone resulted in much weaker effects relative to those of the type-B OsRRs. When we tested combinations of OsHK03, OsHPs, and OsRRs, each combination exhibited distinct CK signaling activities. This system thus allows the rapid and high-throughput exploration of CK signaling in rice.

## 1. Introduction

Plants interact with their environments via the perception of physical and chemical signals, such as light, temperature, and nutrients. Among the multiple mechanisms employed by plants to respond to their surroundings, phytohormones are endogenous chemical signals that take on a prominent role to modulate and coordinate plant growth and development by integrating the external environment and intracellular programs [1,2]. Each phytohormone is recognized by a specific and distinct receptor that then transduces the signal to its downstream cognate signaling components [2,3,4].

Phytohormone signaling cascades are complex due to redundancy among signaling components, which are typically encoded by multigene families, as well as multilayered effects among phytohormones [4]. Thus, a quantitative dissection of the individual contribution of each signaling component has proven difficult because loss-of-function alleles in single genes within a gene family very often cause no obvious alterations in phenotype, thus necessitating the generation of higher-order loss-of-function mutant series to reveal their underlying functions [5,6]. Gain-of-function approaches may be used as an alternative method to characterize the effects associated with individual genes on a given signaling pathway via stable or transient gene overexpression, although the resulting higher levels of the protein under study may not reflect biological functions accurately [7,8].

Transient gene expression systems are powerful methods, as they allow rapid and large-scale screening of signaling components, despite the potential limitations mentioned above. Accordingly, heterologous systems, such as yeast (*Saccharomyces cerevisiae*), and homologous systems for transient gene expression systems, such as protoplasts and *Agrobacterium*-mediated infiltrations, have been used to reconstitute a phytohormone, ABA signaling pathways, and quantitatively measure the function of each signaling component [8,9,10].

Transient gene expression in protoplasts is one of the most popular transient systems for the study of plant signaling pathways [11]. This system consists of a sensitive reporter specific for the signal of interest, in addition to a series of constructs to overexpress individual signaling components in protoplasts, which requires an efficient protoplast isolation and transfection method. Of all the phytohormone cascades, the abscisic acid (ABA) signaling pathway has been the most successfully reconstituted in *Arabidopsis* and rice protoplasts, allowing for a quantitative assessment of the effects of each signaling component [8,9]. However, other phytohormone signaling cascades have yet to be dissected to the same extent as that of ABA.

The phytohormone cytokinin (CK) modulates cell division, senescence, and chloroplast development [12,13]. The CK signaling pathway shares similarity with bacterial two-component signaling (TCS), which transduces various external environmental stimuli to affect gene expression [14,15]. CK signaling has evolved into a more complex system that includes additional signaling intermediates, leading to so-called multistep phosphorelay (MSP) regulation [16]. CK signaling comprises three critical signaling modules, the CK receptors, including histidine kinases (HKs), histidine phosphotransfer proteins (HPs), and type-B response regulators (type-B RRs) [17,18]. CK receptors are transmembrane proteins located at the plasma membrane or the endoplasmic reticulum membrane [19]. The binding of CK to the receptor activates the autophosphorylation activity of the HK at a His residue, which is followed by the transfer of the phosphoryl group to an Asp residue at the C-terminus of the CK receptor. The phosphoryl group is then transmitted to a His residue on the HP, from which it is finally transferred to an Asp residue on type-B RRs in the nucleus [20,21]. Activated type-B RRs induce the transcription of CK-responsive genes by binding to the CK response motif (CRM, 5′-(A/G)GAT(T/C)-3′) present in their promoters [22]. Müller and Sheen reported that a synthetic promoter consisting of multiple copies of the CRM motif driving the expression of the firefly luciferase (*fLUC*) gene acted as a sensitive CK reporter of type-B RR transcriptional activity [23]. This CK reporter was later further optimized, resulting in the new synthetic promoter *TCSn* (*Two Component signaling Sensor new*), which provided an attractive and easy readout of CK signaling in rice and maize (*Zea mays*) when driving the expression of the *Green Fluorescent Protein* (*GFP*) gene in the *TCSn::GFP* reporter [24]. The synthetic *TCSn* promoter was also used to drive the expression of *β-GLUCURONIDASE* (*GUS*) to reveal CK signaling in the root after treatment with 6-benzylaminopurine (BA) [25]. The *TCSn* promoter is therefore an efficient CK sensor that can be combined with different reporter genes to monitor CK signaling in diverse tissues. Recently, a new version of a CK-responsive promoter was also developed, referred to as TCSv2 [26].

In this study, we attempted to reconstitute the CK signaling cascade and identify the effects of each signaling component in rice protoplasts. We transiently cotransfected protoplasts with constructs overexpressing the three CK signaling components (HK, HP, and type-B RR) in addition to the CK sensor *TCSn::LUC* in various combinations. We monitored the activation of CK signaling, as determined by luciferase activity. Our results illustrate the applicability and usefulness of this system to discriminate the contribution of each CK signaling protein in relation to its signaling partners within networks and will be instrumental in deciphering the role of other components of the CK signaling cascade.

## 2. Results

### 2.1. TCSn::fLUC Is a CK-Responsive Reporter in Rice Protoplasts

**Figure 1:**
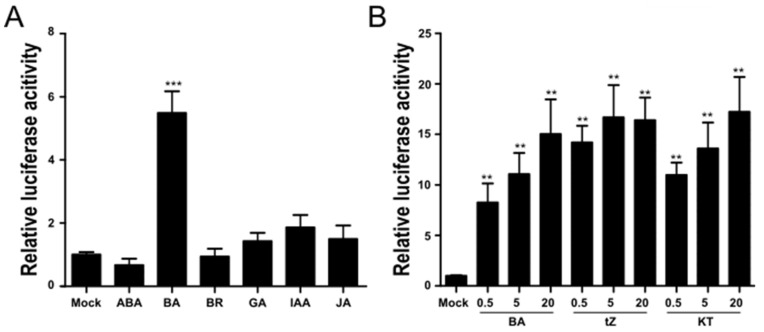
The *TCSn::fLUC* reporter specifically responds to CK in rice protoplasts. (**A**) *TCSn::fLUC* and *OsUbi10::rLUC* constructs were transiently transfected into rice protoplasts using the PEG transformation method. Transfected protoplasts were then exposed to 5 µM ABA, IAA, BR, BA, GA, or JA. Firefly luciferase activity was measured 18 h after transfection. Statistical significance was determined by one-way ANOVA with Tukey’s test, *** *p* < 0.001. (**B**) Rice protoplasts transiently transfected with *TCSn::fLUC* and *OsUbi10::rLUC* were treated with 0.5, 5, and 20 μM of CKs BA, tZ, and KT. *N* = 6. Statistical significance was determined by *t*-test, ** *p* < 0.01. Luciferase activity was measured by comparing with a cotransformed marker, fLUC/rLUC. Values represent averages, and error bars are SEM of three independent biological repeats.

The synthetic *TCSn* promoter consists of 16 copies of the ARR1A motif (5′-NGATT-3′), which is recognized by the type-B RR binding site, and induces the expression of reporter genes in a CK-dependent manner in both *Arabidopsis* and rice [25]. We cloned the *TCSn* promoter into a vector harboring the firefly luciferase gene (*fLUC*) to generate a versatile reporter for CK signaling in rice protoplasts. First, we tested whether this promoter induced luciferase activity specifically in response to CK treatment in rice protoplasts. To this end, we treated transiently transfected rice protoplasts expressing *TCSn::fLUC* and *pUbi10::rLUC* with major phytohormones individually at a final concentration of 5 µM. Only 5 µM BA resulted in a fivefold increase in luciferase activity over mock controls. Other phytohormones failed to raise LUC activity significantly over mock controls, demonstrating the specificity of the *TCSn::fLUC* reporter for CK (Figure 1A). We also tested the *TCSn::fLUC* reporter with other active CKs, kinetin (KT) and trans-zeatin (tZ), together with BA; all three CKs induced LUC activity to the same extent, although BA appeared to be slightly less potent at lower concentrations (Figure 1B). In addition, luciferase activity increased with higher CK concentrations of up to 20 μM (Figure 1B).

### 2.2. Type-B OsRRs Have Different Trans Activities and CK Responsiveness for TCSn Promoter

**Figure 2:**
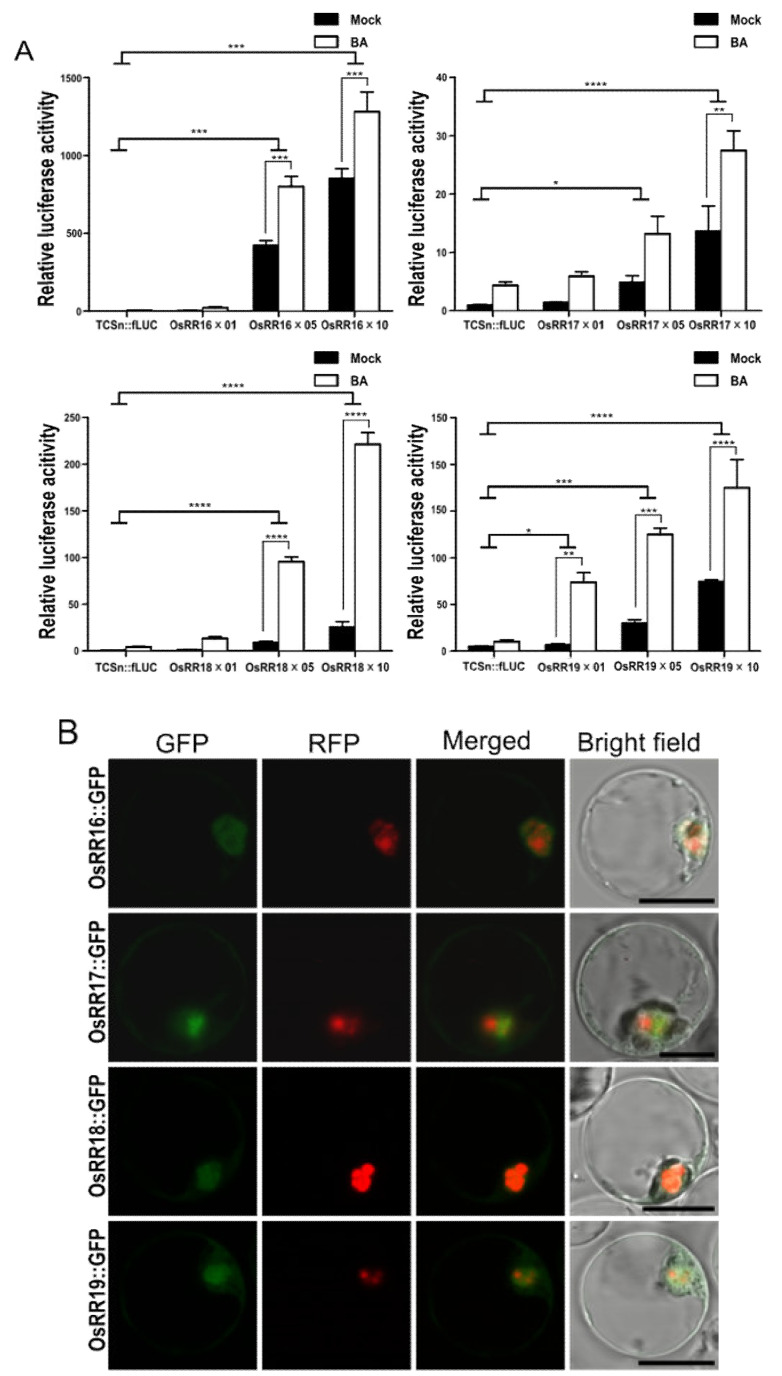
Different transcriptional activations of the *TCSn* promoter by type-B OsRRs in rice protoplasts. (**A**) All type-B OsRRs tested induce the transcription of *fLUC* from the *TCSn* promoter, as indicated by fLUC activity, in an effector DNA concentration-dependent manner. Labels along the *x*-axis (×01, ×05, and ×10) indicate the amount of DNA used for transfection (0.1, 0.5, and 1.0 μg, respectively). Transfected protoplasts were then exposed to 5 µM BA. Firefly luciferase activity was measured 18 h after transfection. Luciferase activity was measured by comparing with a cotransformed marker, fLUC/rLUC. Values represent averages, and error bars are SEM of three independent biological repeats. Statistical significance was determined by two-way ANOVA with Tukey’s multiple comparison test, * *p* < 0.05, ** *p* < 0.01, *** *p* < 0.001, **** *p <* 0.0001. LUC activity measured from mock-treated samples was set to 1. (**B**) Subcellular localization of OsRR16-GFP, OsRR17-GFP, OsRR18-GFP, and OsRR19-GFP in rice protoplasts. NLS-RFP was used as a nuclear marker. Image was magnified by 400×. Scale bar is 10 µm.

Type-B response regulators (RRs) are the MYB transcription factors that bind to the *TCSn* promoter and transcriptionally induce their downstream genes [20]. The *TCSn* promoter, therefore, allowed us to monitor the transcriptional activity of each type-B OsRR in mock-treated or CK-treated protoplasts. Accordingly, we transfected rice protoplasts with *TCSn:fLUC* and effector constructs individually overexpressing the type-B OsRR genes *OsRR16*, *OsRR17*, *OsRR18*, and *OsRR19*. All tested genes strongly induced fLUC activity in an effector DNA dose-dependent manner (Figure 2A). Of all the genes tested here, overexpression of *OsRR16* produced the highest fLUC activity irrespective of BA, and overexpression of *OsRR17* showed the lowest response to BA. In contrast to *OsRR16* and *OsRR17*, the overexpression of *OsRR18* and *OsRR19* resulted in the biggest fold induction of fLUC activity upon BA treatment relative to mock controls (Figure 2A). We determined the subcellular localization of OsRR–GFP fusion proteins to show whether proteins are translated well in protoplasts and have correct localization. All four proteins appeared to accumulate to similar levels, based on fluorescence intensity, and localized to the nucleus, as expected for transcription factors (Figure 2B).

### 2.3. Each HP Protein Increases the Transcriptional Activation of the TCSn Promoter by Type-B OsRRs

**Figure 3:**
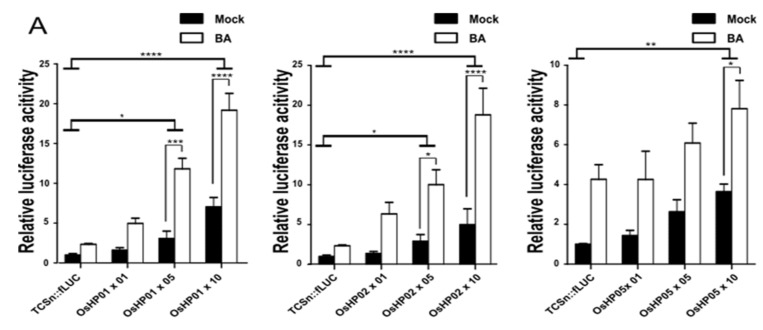

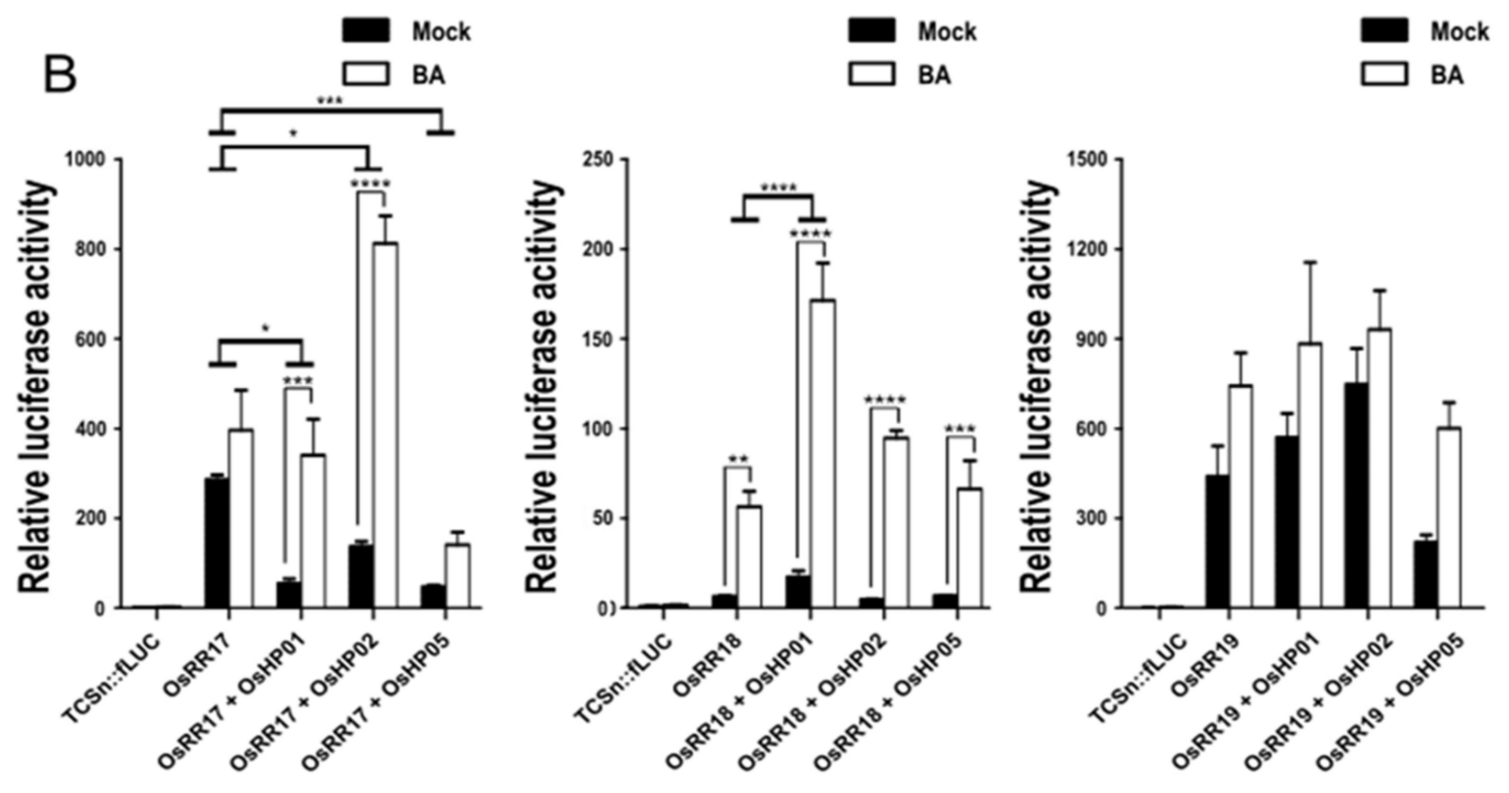
OsHPs modulate the transcriptional activity of type-B OsRRs in rice protoplasts. (**A**) The *OsHP* genes *OsHP01*, *OsHP02*, and *OsHP05* were transiently overexpressed together with the *TCSn::fLUC* reporter. Labels along the *x*-axis (×01, ×05, and ×10) indicate the amount of DNA used for transfection (0.1, 0.5, and 1.0 μg, respectively). (**B**) Effect of cotransfection of OsHPs on the transcriptional activity of type-B OsRRs. *OsRR17*, *OsRR18*, or *OsRR19* and *OsHP1*, *OsHP2*, or *OsHP5* were cotransfected in pairs into rice protoplasts; fLUC activity was measured 18 h after transfection and 5 µM BA treatment. Luciferase activity was measured by comparing with a cotransformed marker, fLUC/rLUC. Values represent averages, and error bars are SEM of three independent biological repeats. Statistical significance was determined by two-way ANOVA with Tukey’s multiple comparison test, * *p* < 0.05, ** *p* < 0.01, *** *p <* 0.001, **** *PC <* 0.0001. LUC activity measured from mock-treated samples was set to 1.

Histidine phosphotransfer proteins (HPs) relay the phosphoryl group from the CK receptor to type-B OsRRs, leading to their activation. The overexpression of *OsHP01* or *OsHP02* alone in rice protoplasts weakly induced fLUC activity, depending on DNA concentration irrespective of CK. By contrast, overexpression of *OsHP05* failed to significantly raise fLUC activity over levels measured for *TCSn:fLUC* alone up to 5 µM DNA concentration (Figure 3A). To monitor the effects of OsHPs for type-B OsRRs, we next cotransfected each *OsHP* with a type-B OsRR as a pair. Notably, *OsHP02* overexpression enhanced the transcriptional activity conferred by *OsRR17* onto the *TCSn::fLUC* reporter. The transcriptional activation of the *TCSn::fLUC* reporter by *OsRR18* increased significantly in response to BA treatment when coexpressed with *OsHP01*. However, overexpression of *OsHP05* had no effect on the transcriptional activation of the *TCSn::fLUC* reporter by type-B OsRRs and appeared to significantly suppress the transcriptional activation of the reporter by *OsRR17* with or without BA treatment (Figure 3B). Notably, OsHPs were not able to increase the reporter activity induced by OsRR19. We also noticed that all three OsHPs reduced the transcriptional activation of the reporter when they were coexpressed with *OsRR17* in the absence of BA; as a result, the specificity of *OsRR17* for BA increased. Because the OsHPs tested here differentially activated OsRRs in our assays and Dortay et al. 2006 reported that HPs are central hubs in interaction among CK signaling components in *Arabidopsis* using yeast two-hybrid assay, we assessed the interaction between OsRR17 and OsHPs by bimolecular fluorescence complementation (BiFC) [27]. All the three combinations tested (OsRR17-VN and OsHP01-VC or OsHP02-VC or OsHP05-VC) showed fluorescence from the Venus variant of GFP, confirming their physical association in planta in the nucleus (Supplementary Materials Figure S1).

### 2.4. The CK Receptor OsHK03 Requires OsHPs to Induce the TCSn:fLUC Reporter

**Figure 4:**
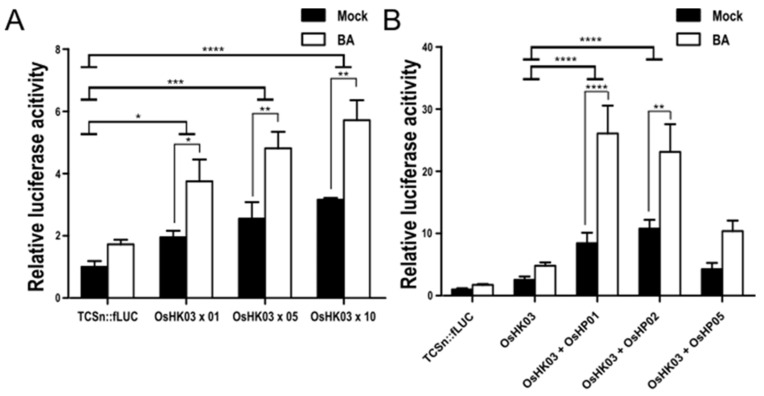
Cotransfection of *OsHK03* and *OsHP*s increases transcriptional activity and sensitivity to BA. (**A**) Overexpression of *OsHK03* weakly activates the *TCSn::fLUC* reporter. Labels along the *x*-axis (x 01, x 05, and x 10) indicate the amount of DNA used for transfection (0.1, 0.5, and 1.0 μg, respectively). (**B**) Transcriptional activation of the reporter by overexpression of *OsHK03* and three *OsHP*s, as measured by dual luciferase assay 18 h after transfection and 5 µM BA treatment. Luciferase activity was measured by comparing with a cotransformed marker, fLUC/rLUC. Values represent averages, and error bars are SEM of three independent biological repeats. Statistical significance was determined by two-way ANOVA with Tukey’s multiple comparison test, * *p* < 0.05, ** *p* < 0.01, *** *p* < 0.001, **** *p* < 0.0001. LUC activity measured from mock-treated samples was set to 1.

The rice and *Arabidopsis* genomes both encode eight histidine kinases (HKs). However, three of them (HK2, 3, 4) are actually CK receptors in *Arabidopsis,* and four of them are functional CK receptors in rice [28,29]. We cloned *OsHK03* and cotransfected the resulting construct with the *TCSn::fLUC* reporter into rice protoplasts. *OsHK03* overexpression only weakly raised fLUC activity over that measured from the reporter alone in an effector DNA concentration- and BA-dependent manner. We then monitored the effect of cotransfecting *OsHP*s and *OsHK03* in protoplasts on reporter activity. Basal transcriptional activation of the reporter significantly increased when *OsHK03* was co-transfected with *OsHP01* or *OsHP02*, but not with *OsHP05*, when compared with fLUC activity from *OsHK03* transfected alone. Upon treatment with BA, the *OsHK03*/*OsHP01* and *OsHK03*/*OsHP02* pairs further increased reporter activity, in sharp contrast to the *OsHK03*/*OsHP05* pair in which reporter activity showed no significant difference with or without BA (Figure 4).

### 2.5. Reconstitution of the CK Signaling Pathway with Type-B OsRR, OsHP, and OsHK in Rice Protoplasts

**Figure 5:**
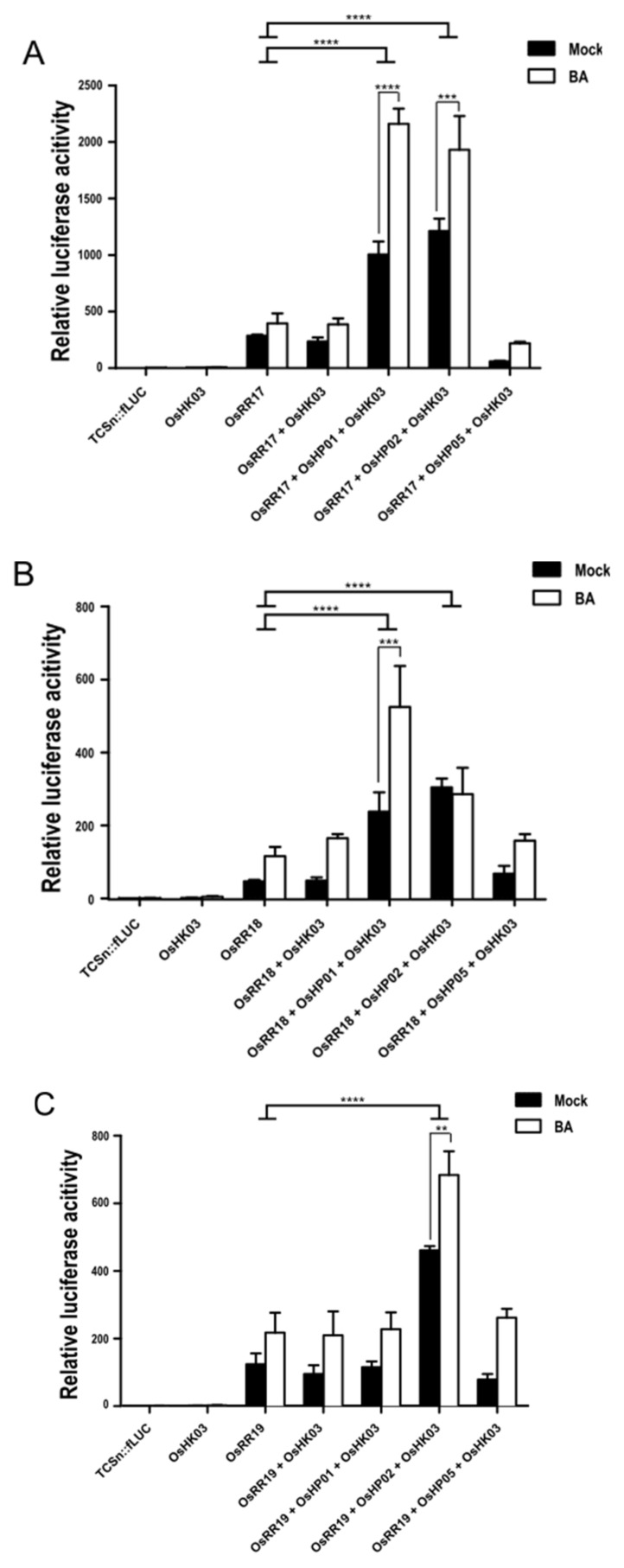
Reconstitution of CK signaling by overexpression of type-B *OsRRs*, *OsHPs*, and *OsHK03* in rice protoplasts. Cotransfection of three transcription factor genes, *OsRR17* (**A**), *OsRR18* (**B**), and *OsRR19* (**C**), with *OsHP*s and *OsHK03* in rice protoplasts. The *TCSn::fLUC* and *pUbi10::rLUC* (a transformation control) reporter constructs were cotransfected with all other effectors. Transcriptional activation of the *TCSn::fLUC* reporter was determined 18 h after transfection and 5 µM BA treatment. Luciferase activity was measured by comparing with a cotransformed marker, fLUC/rLUC. Values represent averages, and error bars are SEM of three independent biological repeats. Statistical significance was determined by two-way ANOVA with Tukey’s multiple comparison test, and error bars indicate SEM, ** *p* < 0.01, *** *p* < 0.001, **** *p* < 0.0001. LUC activity measured from mock-treated samples was set to 1.

Finally, to reconstitute the entire CK transduction cascade in rice protoplasts with the described signaling intermediates cloned here, we transfected the three signaling components, type-B OsRRs, OsHPs, and OsHK alone, in pairs, or altogether (Figure 5). Cotransfection of *OsRR17* with the reporter resulted in a strong induction of fLUC activity that was not BA dependent. The cotransfection of *OsHK03* together with *OsRR17* and the reporter construct did not increase fLUC activity further, suggesting that all three components are required to fully activate CK signaling. Indeed, the cotransfection of *OsHK03*, *OsHP01*, or *OsHP02* and *OsRR17* in rice protoplasts enhanced the basal transcriptional activation of the reporter about three- to fourfold over that seen with OsRR17 alone and conferred sensitivity to BA. As noted earlier with lower-order cotransfections, *OsHP05* failed to activate the reporter when cotransfected with *OsHK03* and *OsRR17* and, in fact, lowered the activity of the reporter (Figure 5A). The overexpression of *OsRR18* with *OsHP01* and *OsHK03* produced results comparable to those seen with *OsRR17*. Cotransfecting *OsRR18* with *OsHP05* and *OsHK03* activated the reporter to the same level as *OsRR18* alone, suggesting that OsRR18 does not participate in signaling with OsHK03 or OsHP05. Finally, cotransfecting *OsRR19* together with *OsHP02* and *OsHK03* was the only combination of constructs that increased reporter activity and exhibited BA sensitivity, as neither OsHP01 nor OsHP05 activated the reporter (Figure 5C).

## 3. Discussion

The major CK signaling components are evolutionarily conserved well across plant species, offering the possibility that the dissection of CK signaling in one plant species will be relevant to all plants. The rice genome encodes four HKs, five HPs (two functional and three pseudo HPs), and 16 type-B RRs. While *Arabidopsis* and rice have similar numbers of type-B RRs and HKs [18], rice is characterized by fewer functional HPs relative to *Arabidopsis* [30].

While many genetic studies have characterized the functions of CK-related genes in *Arabidopsis* and rice [12,23,31], most phenotypes were reported from higher-order mutants to circumvent redundancy; it is therefore difficult to assign a specific function or activity to each gene in CK signaling [32]. The *TCSn* promoter was previously developed and applied to research on CK signaling in *Arabidopsis*, maize, and rice. In maize protoplasts, the *TCSn* promoter conferred a 15-fold induction of the reporter gene when treated with tZ; in rice roots, GUS staining from *TCSn::GUS* reporter lines showed induction about sixfold when exposed to various CKs [23,24,25]. In our rice protoplast system, the *TCSn::fLUC* reporter vector alone displayed an induction around fivefold upon treatment with CK, comparable to the *TCSn::GUS* results in rice roots. Coexpression of the type-B RR genes *OsRR17*, *OsRR18*, and *OsRR19* induced the *TCSn:fLUC* reporter over 100-fold, thus providing a sensitive system in which to monitor the transcriptional activities of type-B RRs and reconstitute the CK signaling cascade. In this transient system, four type-B RRs exhibited distinct activities in terms of CK specificities and transcriptional activation of the reporter. Indeed, OsRR16 and OsRR17 did not activate the reporter in response to CK, whereas OsRR18 and OsRR19 strongly activated the reporter in response to CK. These results suggest that OsRR18 and OsRR19 might have specific functions at various developmental stages or affect tissues or phytohormone levels, which will await experimental validation.

The overexpression of *OsHP* or *OsHK* genes alone failed to significantly induce the activity of the *TCSn::fLUC* reporter. However, the cotransfection of all three components, OsHKs, OsHPs, and type-B OsRRs, significantly increased the reporter activity, supporting the notion that all three components are required for full activation of CK signaling. Endogenous CK signaling components are present in rice protoplasts and activate a CK-responsive reporter to interfere in the dissection of the activity of each signaling component. Heterologous systems, such as yeast or animal cell, can be used to avoid the background noises.

Of the three OsHPs tested here, OsHP05 had very low activity toward the reporter, or even suppressed CK responses. OsHP05 was reported to carry a Glu residue instead of His in the phosphotransfer domain, which might make OsHP5 a pseudo HP [33] (Supplementary Materials Figure S2). This hypothesis would be consistent with our results, as OsHP05 overexpression suppressed CK signaling, possibly via dominant-negative effects. Based on amino acid alignments, rice may therefore only have two functional HPs, OsHP1 and OsHP2, and both increased the transcriptional activity of the reporter significantly when cotransfected with the CK receptor [18]. Notably, OsHP01 and OsHP02 enhanced the transcriptional activation of the *TCSn::fLUC* reporter by OsRR17 in the absence of CK treatment. We interpret this observation as an indication that OsHP increases the CK-specific response of OsRR17. However, other type-B RRs did not show this behavior in relation to OsHPs, possibly because the reconstituted signaling cascade failed to deliver their cognate OsHPs; our conditions may also not have supplied each OsHP with its cognate type-B OsRR. Sun et al. 2014 showed that single knockdown lines for *OsHP01* and *OsHP02* exhibited significant developmental phenotypes during abiotic stress, supporting the notion that these HPs are functional [31]. However, functional differences between the two proteins were not explored, even though our results suggest that OsHP01 and OsHP02 might interact with different type-B OsRRs in terms of signaling network. The CK receptor did not induce CK signaling much when expressed alone or with type-B RRs in *Arabidopsis* or rice protoplasts [14]. We note here that cotransfection of type-B *OsRR*, *OsHP*, and *OsHKs* significantly increased the transcriptional activation of the *TCSn::fLUC* reporter already in the absence of exogenous CK treatment, while CK treatment only increased LUC activity two- to threefold further, suggesting that the endogenous CK content of protoplasts is sufficient to activate CK signaling in our conditions.

Based on the signaling activities observed here, we hypothesize that the OsHK03/OsHP01 and OsHP02/OsRR17 or OsHP02/OsRR18 pairs work alongside the same signaling network because their combination during transfection generated similar signaling activity. By contrast, OsRR19 does not appear to engage in a significant interaction with OsHP01, as its cotransfection with *OsHP01* and *OsHK03* weakly activated the reporter construct.

Plant signaling is very complex and relies on multilayered interactions. A better understanding can be obtained via bioinformatics analysis of gene families and gene expression profiles in response to genetic perturbations, from physical interaction networks, yeast two-hybrid assays, and pull-downs, as well as from proteomics and metabolomics, which require thorough, time-consuming integration. In this work, we aimed to reconstruct individual branches of CK signaling to dissect the effects of each component. The protoplast system allows high-throughput analysis in planta and shows low noise compared with whole plants. Luciferase activity can be easily monitored from transfected protoplasts and constitutes a very powerful tool to study CK signaling and inform candidate gene identification and analysis.

## 4. Materials and Methods

### 4.1. Plant Materials and Growth Conditions

The rice (*Oryza sativa*) cultivar Dongjin was used in this study. Hulled rice seeds were surface-sterilized with 70% ethanol for 30 s, followed by 50% NaClO containing a drop of Tween-20 for 15 min with constant agitation and five washes with distilled water. The NaClO sterilization step was repeated once but without Tween-20. The surface-sterilized seeds were thoroughly dried using sterilized dry filter paper. The seeds were then sown on half-strength Murashige and Skoog (1/2 MS) medium (supplemented with 0.4% Phytagel and adjusted to pH 5.8) and allowed to germinate for 10 d in the dark and 2 d in the light at 28 °C before protoplast isolation.

### 4.2. Isolation of Rice Protoplasts

To isolate protoplasts from young rice seedling, shoots were sliced into 1 mm strips and incubated in enzyme solution (1.5% Cellulase R-10 (Yakult Pharmaceutical Inc., Tokyo, JP), 0.75% Macerozyme R-10 (Yakult Pharmaceutical Inc., Tokyo, JP), 0.6 M mannitol, and 10 mM 2-(N-morpholino)ethanesulfonic acid (MES), pH 5.7 adjusting with KOH) that had been activated at 65 °C for 20 min. The mixtures containing rice tissue and enzymes were incubated by shaking for 4 h at 28 °C, filtered on a 145 μm mesh, and diluted with five volumes of W5 solution (154 mM NaCl, 125 mM CaCl_2_, 5 mM KCl, 5 mM glucose, and 3 mM MES, pH 5.7 adjusting with KOH). The diluted cell suspension was centrifuged at 100× *g* for 10 min at room temperature. The protoplast pellet was then resuspended in 3 mL of W5 solution and placed on top of 23% sucrose and centrifuged at 100× *g* for 10 min to remove remaining debris. The volume of separated protoplasts was then adjusted to 10 mL with W5 solution, and the cell density was determined on a hemocytometer. The protoplasts were centrifuged at 100× *g* for 7 min at room temperature and resuspended in MaMg solution (600 mM mannitol, 15 mM MgCl_2_, and 5 mM MES, pH 5.7 adjusting with KOH) for transfection.

### 4.3. Transient Gene Expression in Rice Protoplast

Protoplasts (4–5 × 10^4^ cells) were suspended in 300 µL MaMg solution and mixed with up to 30 μg of DNA and 40% PEG solution (400 mM mannitol, 100 mM Ca(NO_3_)_2_, and 40% PEG 6000). The solution was mixed thoroughly using a fixed-angle rotating wheel for 10 min and incubated for 30 min at room temperature. After incubation, 630 µL, 1.25 mL, and 2.5 mL of W5 solution were added every 10 min over the course of 30 min. The mixtures were incubated for another 25 min and centrifuged at 100× *g* for 5 min to remove the PEG solution. The protoplast pellets were resuspended in 3 mL of W5 solution and incubated at 28 °C overnight (18 h) with or without CKs in the dark.

### 4.4. Construction of Vectors

We selected the synthetic Two Component signaling Sensor new (*TCSn*) promoter as a reporter in rice, as it specifically responds to CK [24]. The *TCSn* promoter was cloned upstream of the firefly luciferase (*fLUC*) gene to generate a CK reporter. The coding sequences for various CK signaling components (type-B response regulator (RR), histidine phosphotransfer protein (HP), and histidine kinase (HK)) were PCR-amplified, cloned into the pENTRY D-Topo vector (Invitrogen, Carlsbad, CA, USA), and then recombined into destination vectors for signaling effectors, subcellular localization, and bimolecular fluorescence complementation (BiFC) assays by LR recombination (Invitrogen, Carlsbad, CA, USA) as previously described [8]. The primers used are listed in Supplementary Materials Table S1.

### 4.5. Dual Luciferase Assays

For dual luciferase assays, *TCSn:fLUC*, *Renilla* luciferase driven by the ubiquitin promoter (*pUbi10::rLUC*), and effector constructs were introduced into rice protoplasts by PEG-mediated transfection. After 18 h of incubation, the protoplasts were centrifuged at 300 g for 30 s and the supernatants removed. Pelleted cells were resuspended in 50 µL 1× passive lysis buffer (PLB). *Renilla* and firefly luciferase activities were measured with 10 µL lysate according to the manufacturer’s protocol (Promega, Madison, WI, USA); firefly luciferase activity was then normalized to *Renilla* luciferase activity to account for transfection efficiency.

### 4.6. Subcellular Localization and Bimolecular Fluorescence Complementation (BiFC) Assay

To determine the localization of type-B OsRRs in plant cells, we transfected rice protoplasts with *OsRR–GFP* fusion constructs. Red fluorescent protein (RFP) with a nuclear localization sequence (NLS) was used as nucleus-localized control. GFP fluorescence (493–546 nm) was observed and imaged using a Leica TCS SP8 confocal microscope (Leica Microsystems, Germany). For BiFC assays, OsRR17 was fused to the N-terminus of the Venus variant of GFP (OsRR17-VN), while three OsHPs were fused to the C-terminus of Venus (OsHP-VC). The corresponding constructs were transfected in rice protoplasts as above. *OsbZIP10-VC* was used as a negative control, and NLS-RFP was used as a nucleus-localized control. RFP fluorescence (581–652 nm) was observed and imaged using a Leica TCS SP8 confocal microscope.

## Supplementary Materials

The following are available online at https://www.mdpi.com/article/10.3390/ijms22073647/s1.
