# Supplementary material for: Reconstitution of Cytokinin Signaling in Rice Protoplasts
Source: Int J Mol Sci. 2021 Mar 31;22(7):3647. doi: 10.3390/ijms22073647 (PMC8037374; doi:10.3390/ijms22073647)
Supplement: Supplementary file 1 [file ijms-22-03647-s001.pdf]

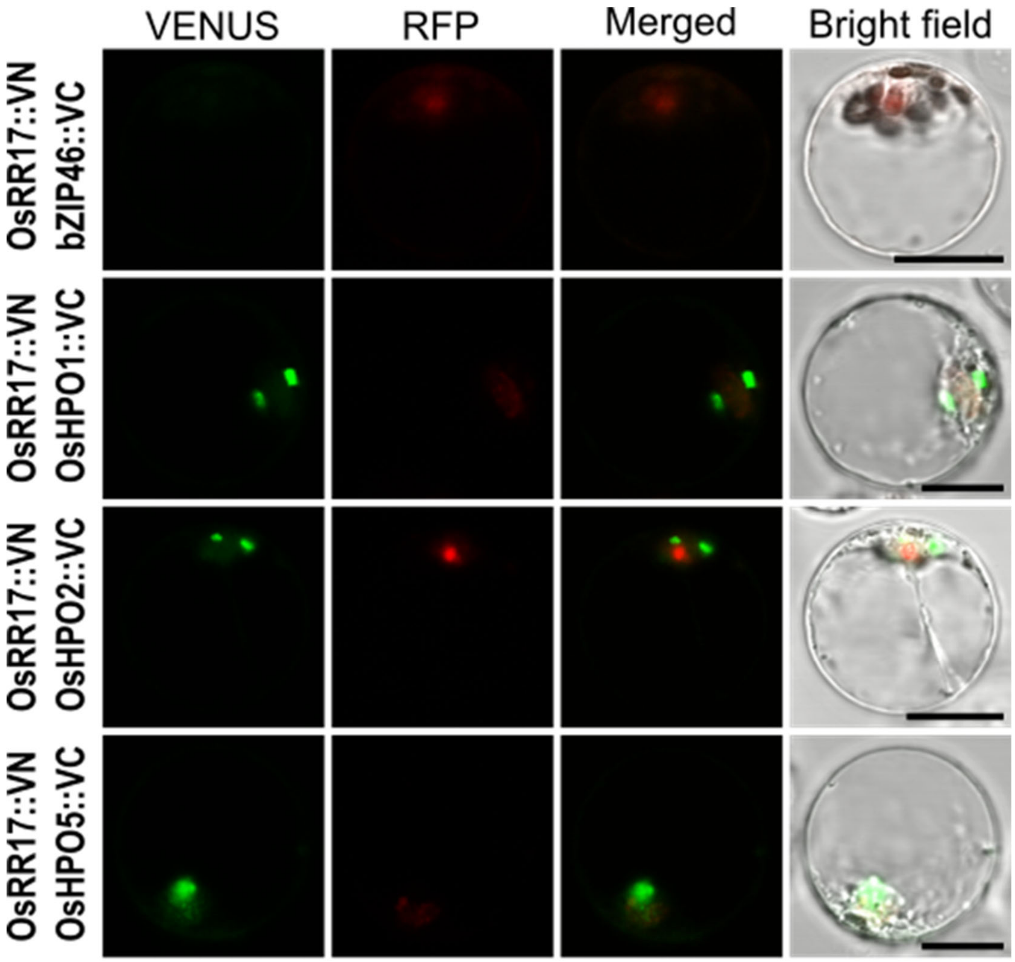

Figure S1. Bimolecular fluorescence complementation (BiFC) analysis of the interaction between OsRR17 and OsHPs**.** The potential interactions between OsRR17 and three OsHPs (OsHP01, OSHP02, and OsHP05) were tested by BiFC. NLS-RFP was used as a nuclear marker. Bar = 10 μm.

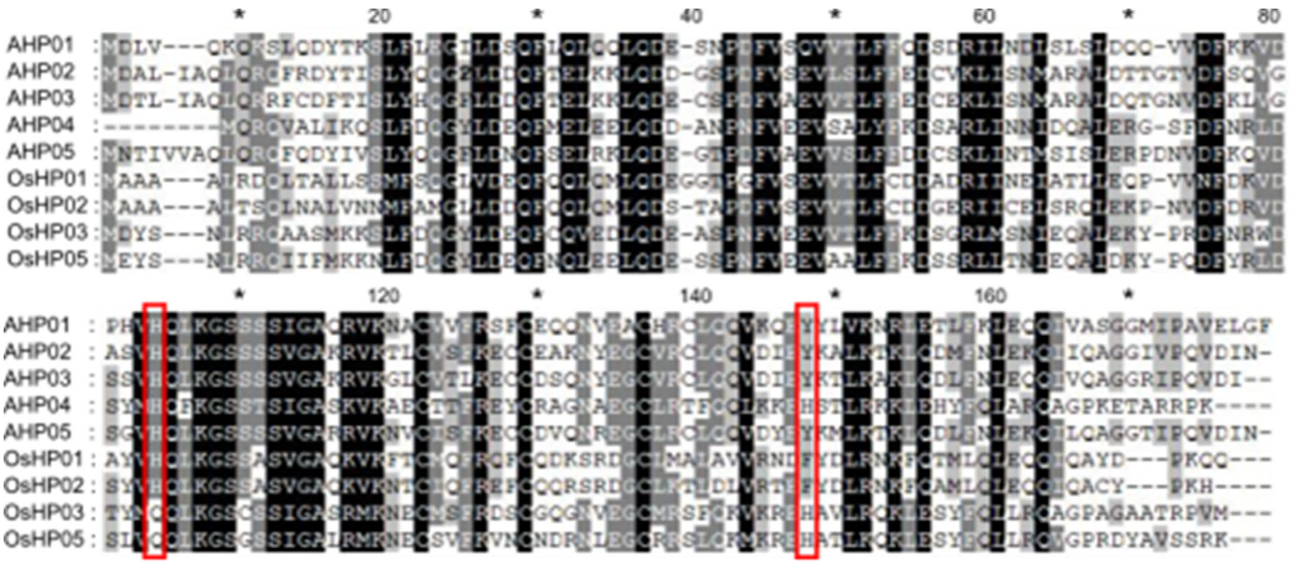

Figure S2. Amino acid alignments of OsHPs and comparison of the phosphotransfer domains, highlighted by the red boxes.

Table S1. Primer sequences used in this manuscript

Transcript	Locus #	CDS length (bp)	Direction	Sequence
OsHK03	*Os01g69920*	3072	Forward	caccATGGATGAGATGAGCTGCG
			Reverse	CTATTCAACTTGGTCATGA
OsHP01	*Os09g39400*	450	Forward	caccATGGCGGCCGCCGCTCTCC
			Reverse	CTATTCAACTTGGTCATGA
OsHP02	*Os08g44350*	444	Forward	caccATGGCGGCCGCCGCGCTG
			Reverse	TTAATGTTTAGGGTAACAAG
OsHP05	*Os05g09410*	456	Forward	caccATGGAGTATTCAAATTTGCG
			Reverse	TTACTTCCTTGAGCTCACTG
OsRR16	*Os01g67770*	1749	Forward	caccATGGACGCCACCGCCTTCC
			Reverse	TCAGGATGATGCAAAGAGAC
OsRR17	*Os02g08500*	1881	Forward	caccATGACGGTGGAGGAGAGGC
			Reverse	CTAGACCAGCTCCCAGTCCC
OsRR18	*Os02g55320*	2067	Forward	caccATGAGGGCGGCGGAGGAGAG
			Reverse	TCATATGCAAGCTCCAAGG
OsRR19	*Os03g12350*	2076	Forward	caccATGGCGCCGGTGGAGGATG
			Reverse	TCACATCTGTCCACTAAATCC
